# Screening core genes and signaling pathways after SFTSV infection by integrated transcriptome profiling analysis

**DOI:** 10.1016/j.virusres.2023.199138

**Published:** 2023-05-23

**Authors:** Huimin Fu, Yanhong Wang, Chuanfei Yuan, Yuhang Zhang, Aihua Zheng, Zhen Zou, Qianfeng Xia

**Affiliations:** a Key Laboratory of Tropical Translational Medicine of Ministry of Education, NHC Key Laboratory of Tropical Disease Control, School of Tropical Medicine, The Second Affiliated Hospital, Hainan Medical University, Haikou, Hainan, 571199, China; b State Key Laboratory of Integrated Management of Pest Insects and Rodents, Institute of Zoology, Chinese Academy of Sciences, Beijing 100101, China; c CAS Center for Excellence in Biotic Interactions, University of Chinese Academy of Sciences, Beijing 100049, China

**Keywords:** Severe fever with thrombocytopenia syndrome virus (SFTSV), RNA-Seq, Transcriptome analysis, Virus-host interaction

## Abstract

A newly discovered tick-borne virus called the severe fever with thrombocytopenia syndrome virus (SFTSV) can cause the severe fever with thrombocytopenia syndrome (SFTS). The mortality and incidence rate of SFTS patients remain extremely high due to the fast global dissemination of its arthropod vectors, and the mechanism of viral pathogenesis remains largely unknown. In this study, high-throughput RNA sequencing (RNA-Seq) was used to sequence HEK 293 cells treated with SFTSV at four time points. 115, 191, 259, and 660 differentially expressed genes (DEGs) were identified at 6, 12, 24, and 48 h post-infection, respectively. We found that SFTSV infection induced the expression of genes responsible for numerous cytokine-related pathways, including TNF, CXCL1, CXCL2, CXCL3, CXCL8, CXCL10, and CCL20. With the extension of infection time, the expression of most genes involved in these pathways increased significantly, indicating the host's inflammatory response to SFTSV. Moreover, the expression levels of GNA13, ARHGEF12, RHOA, ROCK1, and MYL12A, elements of the platelet activation signaling pathway, were downregulated during SFTSV infection, suggesting that the SFTSV infection may cause thrombocytopenia by inhibiting platelet activation. Our results contribute to further understanding the interaction between SFTSV and the host.

## Highlights

• RNA-seq measures gene expression of SFTSV-infected HEK 293 cells.
• SFTSV infection induces upregulation of genes in many cytokine-related pathways.
• By preventing platelet activation, SFTSV infection may cause thrombocytopenia.
• Hub genes revealed by the PPI network of 48 h will help with clinical research.

## 1 Introduction

A new Dabie *bandavirus* in the *Phenuiviridae* family, the severe fever with thrombocytopenia syndrome virus (SFTSV) is spread by ticks (Yu et al., 2011). *Haemaphysalis longicornis* and *Dermacentor silvarum* are the most common ticks that transmit SFTSV to humans and animals (Luo et al., 2015; Hu et al., 2020). Previous studies have shown that patient bodily fluids such as blood, saliva, and tears can transmit SFTSV (Liu et al., 2012; Sun et al., 2021). The main clinical symptoms of severe fever with thrombocytopenia syndrome (SFTS) are high fever, thrombocytopenia, leukopenia, and multiple organ dysfunction (Yu et al., 2011; Liu et al., 2013). Since the first SFTS case was reported in 2009, the incidence rate has expanded to at least 23 provinces in China (Yu et al., 2011; Zhan et al., 2017). The epidemic appears to be spreading in Korea, Japan, and Vietnam (Kim et al., 2013; Takahashi et al., 2014; Tran et al., 2019). However, we still do not have effective treatments or vaccines against SFTSV.

The genome of SFTSV contains large (L), middle (M), and small (S) segments. The viral RNA-dependent RNA polymerase (RdRp) is encoded by the L segment, which is responsible for initiating viral RNA replication and transcription. The viral glycoprotein N (Gn) and glycoprotein C (Gc) are encoded by the M segment and are responsible for the formation of heterodimers of viral particles and mediating viral attachment to cells (Elliott and Brennan 2014; Lei et al., 2015). A non-structural protein (NSs) and a nucleoprotein (NP) are both encoded by the S segment in the antisense direction (Zhou et al., 2013). NSs is responsible for mediating genome replication and virion assembly (Ning et al., 2014; Khalil et al., 2021).

SFTSV relies on the envelope protein Gn/Gc to adhere to the cell surface and transport viral particles into the cell by membrane fusion (Tani et al., 2016; Yuan and Zheng 2017). Previous studies have reported that SFTSV glycoprotein binds to cell surface receptors such as DC-SIGN, NMMHC-IIA, and HS, then undergoes endocytosis through the action of calreticulin and clathrin to promote virus entry into cells; Gc is thus considered to be a key membrane fusion protein for SFTSV infection (Lozach et al., 2011; Sun et al., 2014; Halldorsson et al., 2016; Kim et al., 2019). The host will stimulate the related immune responses to resist SFTSV infection. The IFN response or pro-inflammatory response acts as the host's first line of defense against viral infection. These not only play critical roles in the elimination of initial virus replication but also aid in the stimulation of adaptive immunity (Koyama et al., 2008; Mogensen 2009; Takeuchi and Akira 2009). Recently, it was reported that SFTSV infection activates RIG-I and TLR3 that can recruit MAVS and stimulate signal transduction, further activating interferon regulatory factors (IRFs) and nuclear factor -κ-gene binding (NF-κB) to produce IFNs. IFNs activate ISG transcriptional activity and inflammatory cytokine production by binding to the interferon a/b receptor (IFNAR), establishing the host's antiviral response (Santiago et al., 2014; Chaudhary et al., 2015; Hong et al., 2019; Min et al., 2020). However, viruses have developed mechanisms to get around the host's defenses. Most studies have shown that the innate immune evasion mechanism of *Phenuiviridae* is associated with NSs, which can inhibit the interferon regulatory factor 3 (IRF3) to stop the initiate transcription of interferon genes. NSs also interact with RIG-I and the E3 ubiquitin ligase TRIM25, rendering them inactive for the induction of interferon gene transcription (Ning et al., 2014; Mendoza et al., 2019; Liu et al., 2020). The pro-inflammatory responses must be balanced with induced anti-inflammatory responses, as excessive pro-inflammatory activation may be a critical factor in disease severity (Basler 2017). In addition, thrombocytopenia is an important clinical symptom in patients with SFTS, but the primary mechanism of thrombocytopenia is not clear. Related studies have reported that bone marrow aspirates from SFTS patients exhibit hemophagocytic syndrome (HLH) caused by viral infection, characterized by phagocytosis of platelets and lymphocytes (Takahashi et al., 2014; Fill et al., 2017; Kim et al., 2018).

RNA sequencing (RNA-seq) technology is currently a common method for analyzing gene expression and discovering new RNA (Wang et al., 2009). It will help to clarify the host systemic response induced by viral infection, which is critical for a comprehensive understanding of the virus's life cycle and pathogenicity. In this study, to investigate the host response induced by SFTSV infection, we performed the time-course transcriptome analysis of virus infected human embryonic kidney 293 (HEK 293) cells, which are susceptible to SFTSV infection, and previous studies have shown that SFTSV infection can lead to kidney cell lesions in mice and other animals (Liu et al., 2014; Drake et al., 2017). The differentially expressed genes (DEGs) were identified through RNA-seq and determined the signal pathways related to the SFTSV infection. This study can help determine the SFTSV–host interactions and thereby provide a theoretical basis for further research.

## 2 Results

### 2.1 Transcriptome analysis revealed the general gene expression profile of HEK 293 cells infection with SFTSV

**Fig. 1:**
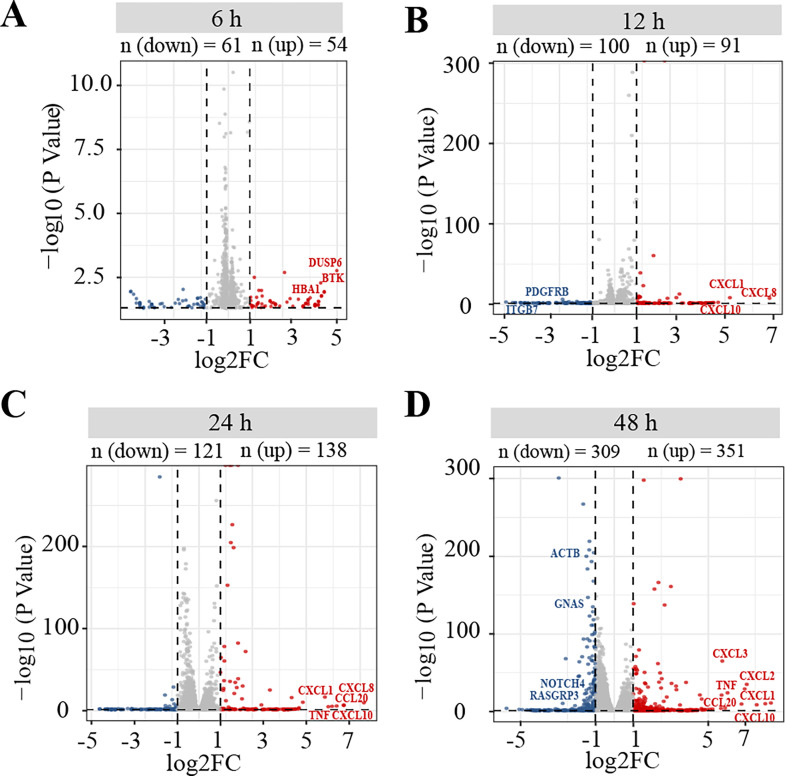
Volcano plot of the differently expressed genes (DEGs) The x-axis is the log2 scale of the fold change of gene expression (log2(Fold Change)) at different times after SFTSV infection (A-D, SFTSV vs MOCK 6, 12, 24, and 48 h). Negative values indicate downregulation; positive values indicate upregulation. The y-axis is the minus log10 scale of the P-value (–log 10(P-value)) that indicates the significance level of the expression difference. The red dots represent significantly upregulated genes, while the blue dots represent significantly downregulated genes, and the gray dots indicate non-significant differentially abundant genes. The most differentially expressed genes were labeled in the plot.

After SFTSV infection at four different time points, we used RNA-seq to analyze the gene expression profiles. SFTSV was added into HEK 293 cells at a multiplicity of infectivity (MOI) of 5, and untreated cells (MOCK) were used as controls. It showed that the HEK 293 cells were in a normal process of infection process (Figure S1A) (Deng et al., 2012). Total RNA was isolated from the collected cells at 6, 12, 24, and 48 h following infection. We firstly detected the mRNA levels of Gn and Gc at different time post-infection by quantitative real-time PCR (qRT-PCR) to monitor viral infection. As compared with MOCK, the results showed that Gn and Gc expression levels gradually elevated over the course of the infection. The mRNA level of Gn peaked at 24 h after infection, being upregulated about 6-fold, and Gc peaked at 48 h after infection, upregulated about 4-fold (Figure S1B), indicating that SFTSV successfully infected and replicated in the cells. Next, the different groups of samples were sequenced on the Illumina NovaSeq™ 6000 platform, and the fold changes (FC) of each gene at different time points were calculated by DESeq2 software. The Principal Component Analysis (PCA) showed that each group of three replicates exhibits significant consistency (Figure S1C). The results showed that a total of 9500 host genes were quantified using a threshold P-value of 0.05. Compared with MOCK, a total of 1145 DEGs, defined as P-value ≤ 0.05 and log2FC ≥ 1, were identified (Table S1). Volcano plots showed that different numbers of DEGs were identified at the four time points of infection (Fig. 1A–D). At the initial stages of infection (6 and 12 h), only a few DEGs were found, 115 and 191, respectively. Among them, DUSP6, BTK, CXCL1, and CXCL8 were significantly upregulated, while ITGB7 and PDGFRB were significantly downregulated (Fig. 1A and B). The number of DEGs dramatically increased in the later stages of infection (24 and 48 h). At 24 h after infection, there were 259 DEGs, and at 48 h, there were 660 DEGs, which was more than the combined number of the previous three time points (Fig. 1C and D). Similar to 12 h, genes significantly upregulated in the later stages of infection were cytokines or chemokines, such as CXCL1, CXCL2, CXCL3, CXCL8, CXCL10, and CCL20 (Fig. 1B–D). These findings collectively suggested SFTSV infection caused the strongly inflammatory and cytokine storm (Li et al. 2021; Seo et al. 2021; Zhou and Yu 2021). Many solute carrier (SLC) family genes were also activated at 24 or 48 h after infection, including SLC16A12, SLC2A3, SLC16A6, and SLC32A1. This is consistent with previous findings showing that viral infection affects ion channels of biomembrane (Table S1) (Yan et al., 2021). At 48 h, we also found that some genes were significantly downregulated, including ACTB, GNAS, and RASGRP3 (Fig. 1D).

In addition, we analyzed the temporal clustering of 1145 DEGs by Mfuzz. Eight distinct clusters of gene expression trend were identified over time of infection (Figure S1D, Table S2). For example, genes involved in clusters 1 and 8 had similar expression patterns, and the upward trend of these clusters was particularly clear, that is, they showed upward trends with the time of infection with SFTSV. Genes involved in cluster 7 showed a downward trend with the extension of infection time, which is consistent with previous studies showing that SFTSV infection aids viral replication by inhibiting related signaling pathways (Park et al., 2021). While genes involved in clusters 2–6 showed complex change trends (Figure S1D). These results revealed the synergistic effects of DEGs during different periods of viral infection; such genes displaying clear trends will help us to analyze the biological response induced by viral infection.

### 2.2 GO enrichment analysis of DEGs

**Fig. 2:**
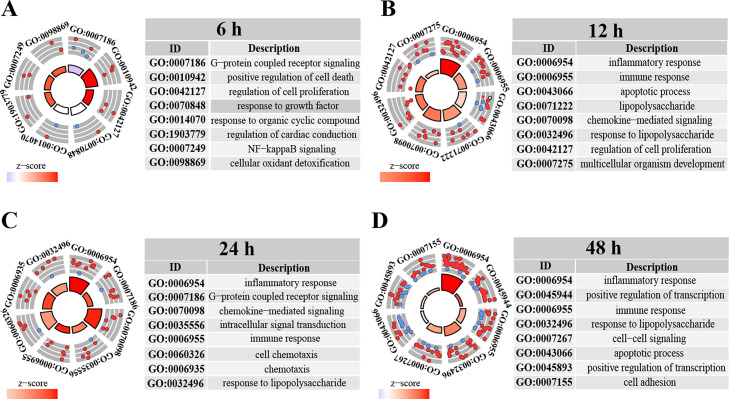
GO enrichment analysis of the DEGs based on biological processes The DEGs of four time points were analyzed with the DAVID database for determining which differentially regulated genes were involved in biological processes; these are shown by a turquoise module genes plot (A–D, 6, 12, 24, and 48 h). The red spots represent the upregulated genes, and the blue spots represent the downregulated genes. The center of the circle indicates the gene cluster degree from a cluster analysis based on the Z-score (standard score).

GO analysis of DEGs at different time points was performed to determine categories. The top five elements of molecular function (MF), cellular composition (CC), and biological process (BP) are showed in Figure S2. The result showed that at 6 h after SFTSV infection, the upregulated DEGs aggregated in BP, such as cell death and regulation of cell proliferation, while the MF clustered lots of downregulated DEGs, including collagen binding (Figure S2A). At 12 h, the DEGs were enriched in MF, such as chemokine activity, cell junction, and ATF6-mediated UPR (Figure S2B). This suggests that SFTSV infection can activate the unfolded-protein response (UPR) to relieve the endoplasmic reticulum (ER) stress (Chan 2014; Zhang et al., 2019). The upregulated DEGs were markedly enriched in BP at 24 and 48 h, including inflammatory response and response to lipopolysaccharide (Figure S2C and D). Furthermore, various downregulated DEGs most belong to mitochondrial electron transport and cytochrome-c oxidase activity, were considerably concentrated in BP and MF at 48 h (Figure S2D). To characterize the functions of DEGs involved in SFTSV infection, we further used a turquoise modular gene plot to analyze the DEGs of the top 8 BP terms at four time points. The results revealed that the upregulated DEGs at 6 h post-infection was primarily clustered in the positive regulation of cell death and regulation of cell proliferation, whereas the G-protein coupled receptor signaling pathway contained most downregulated DEGs (Fig. 2A). Upregulated DEGs at 12 and 24 h post-infection was significantly enriched in inflammatory response and chemokine-mediated signaling (Fig. 2B and C). Moreover, the downregulated DEGs were concentrated in the apoptotic process at 48 h post-infection, whereas the upregulated DEGs were markedly concentrated in inflammatory response, response to lipopolysaccharide, and cell-cell signaling (Fig. 2D).

### 2.3 Enrichment analysis of KEGG pathway of DEGs

The top five pathways were then examined using gene set enrichment analysis (GSEA) at four different time points following SFTSV infection. The results showed that at 6 h, the gene set was enriched in the ribosome (Figure S3A), while at 12 h, the gene set was highly enriched in four signaling pathways associated with cytokines, i.e., chemokine signaling, TNF signaling, IL-17 signaling, and RIG-I-like receptor signaling (Figure S3B). The gene sets at 24 and 48 h were also enriched in the NF-κB signaling pathway compared with 12 h (Figure S3B–D). We also found that as the time of infection progressed, the enrichment score (ES) was highest at 24 h after infection, indicating that the host response was strongest at 24 h (Figure S3C). Each of these signaling pathways was closely associated with the host cytokine response.

**Fig. 3:**
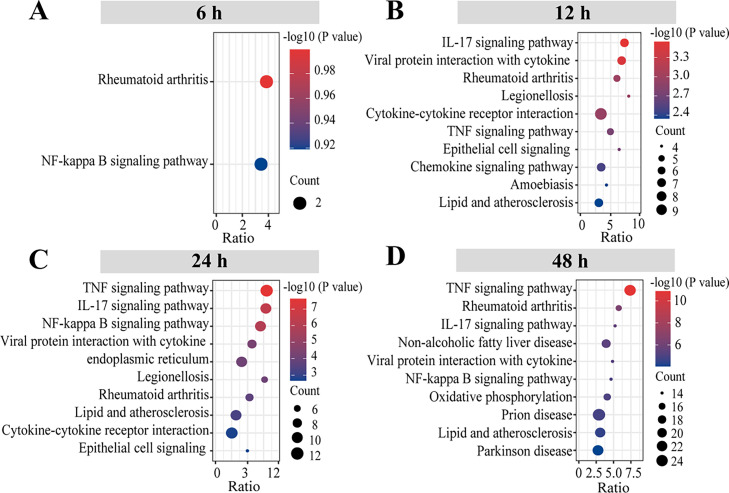
KEGG enrichment analysis of the DEGs The top 10 enriched KEGG pathways of DEGs at four time points (A–D, 6, 12, 24, and 48 h) after SFTSV infection. The y-axis represents KEGG-enrichment terms, and the x-axis represents the ratio of the number of genes in the pathway to the total number of genes located in the pathway. The bubble indicates the number of genes enriched in a particular pathway. Red to blue indicate minus log10 P-value (–log10 (P-value)).

To further illustrate the distinct pathways of DEGs involved in SFTSV at various time points, we carried out KEGG pathway analysis. The study found that at 6 h post-infection, the DEGs were primarily enriched in two signaling pathways, Rheumatoid arthritis (two genes) and NF-κB signaling pathway (two genes) (Fig. 3A). At 12, 24, and 48 h, DEGs were enriched in more pathways compared with 6 h, including IL-17 signaling pathway, TNF signaling pathway, viral protein interaction with cytokines, and cytokine–cytokine receptor interaction, most of which were involved in inflammatory responses (Fig. 3B–D).

### 2.4 Expression of genes involved in immune response against SFTSV

We discovered that the majority of the DEGs were considerably enriched in NF-κB and cytokine-associated signaling pathways during SFTSV infection based on the aforementioned GO and KEGG analyses. Furthermore, related studies have shown that NF-κB and cytokines were significantly involved in host-related immune pathways (Sun et al., 2021; Zhou and Yu 2021). A threshold P-value of 0.05 was used to identify 404 genes implicated in the NF-κB and cytokine signaling pathways (Table S3), and the expression profiles of these genes are shown in Figure S4. According to the results of the hierarchical cluster analysis, in the NF-κB signaling pathway, the genes of cluster I rarely changed in the early stages of infection (6 and 12 h), but were significantly downregulated in the late stages of infection (24 and 48 h). The genes in cluster II were upregulated during the later stages (Figure S4A). In the expression profiles of genes involved in cytokine signaling pathways, we found that the genes of cluster I were downregulated after infection and more significantly at later stages, while the genes of cluster II were significantly upregulated at later stages of infection (Figure S4B).

**Fig. 4:**
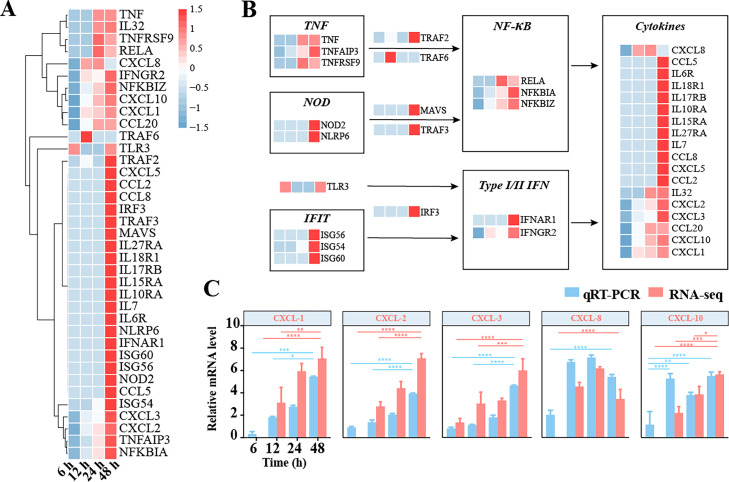
Expression profile of genes involved in cytokines signaling The graph shows a total of 37 selected factors involved in cytokines response to SFTSV infection based on the clustering of similar expression profiles (A) and in topological context (B), and each grid represents the log2FC of the selected genes. From left to right represent different time points of infection, being 6, 12, 24, and 48 h. Comparison of RNA-seq-based and qRT-PCR-based relative expression levels for selected cytokine genes, *n* = 4 (C). Error bars indicate SEM. Asterisks indicate significance level after multiple test correction (false discovery rate; FDR): *P < 0.05; **P < 0.01; ***P < 0.001; ****P < 0.0001.

Based on the above expression profiles analysis, we identified a total of 37 DEGs from cluster II in the NF-κB and cytokine signaling pathways, and clustered the relative expressions of these DEGs at different time points (Table S4, Fig. 4A). The results showed at 48 h after SFTSV infection, the expression of most genes was significantly higher than that at other times. In addition, we further investigated the expression patterns of these genes through topological plots. We discovered that type I/II IFN and NF-κB signaling pathways regulated the expression of 18 cytokine genes. (Table S4, Fig. 4B). Taking a close look at these results, we found that the activated TNF signaling pathway mediated the activation of NF-κB via TRAF2 and TRAF6, and the activation of the NOD signaling pathway activated the NF-κB signaling pathway by inducing MAVS and TRAF3 (Munroe and Bishop 2004; Li and Zhu 2020; Yang and Shu 2020). Genes involved in the TLR3 and interferon-stimulated gene 56 families, both of which are heavily increased at post-infection, will mediate cytokine production by regulating transcription of type I/II IFNs (Fensterl and Sen 2011; Schneider et al., 2014). For example, TNF, CXCL10, and CCL20 were specifically upregulated in clinical laboratory data from SFTS patients as previously reported (Fig. 4B) (Li et al., 2021; Park et al., 2021; Wang et al., 2021). Moreover, we randomly selected some cytokines, such as CXCL1, CXCL2, CXCL3, CXCL8, and CXCL10, and measured their expression levels by qRT-PCR. The outcomes demonstrated that the expression patterns of these genes were consistent with the results of RNA-seq (Fig. 4C). The initiation of these signaling pathways that regulate cytokines indicates that the host has activated many cytokines for resistance to viral infection, and that these cytokine storms may be a critical factor in the death of SFTS patients.

### 2.5 Identification of expression profiles of genes involved in platelet activation-related pathways

**Fig. 5:**
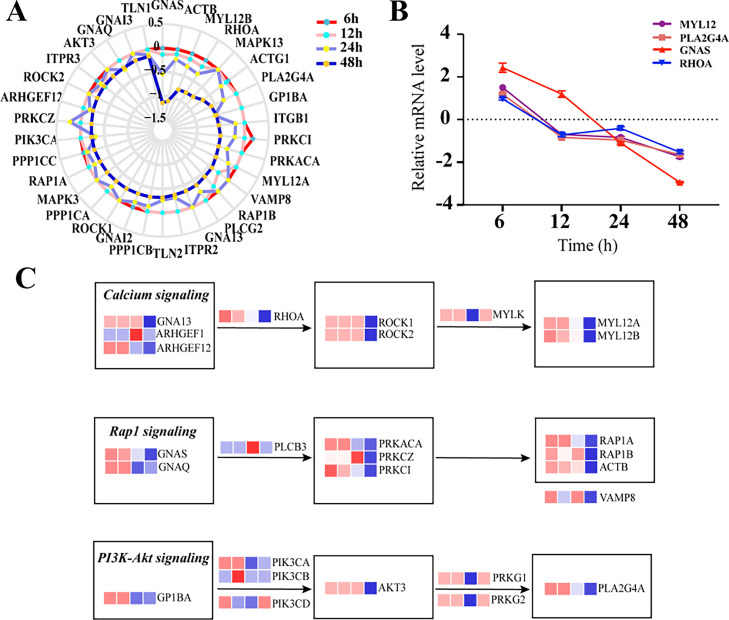
Detailed expression analysis of genes involved in platelet activation signaling pathway (A) Radar plot of the mRNA expression levels of 34 genes involved in the platelet activation pathway. The different colored lines represent 6, 12, 24, and 48 h. (B) Determination of relevant genes involved in the platelet phase pathway using qRT-PCR and illustrated by a line graph. Actin was chosen as an internal control. Intracellular RNA levels at each time point of SFTSV infection were normalized to those in the MOCK cells. All experiments were performed at least three times, and values represent the means and SEM from three replicates. (C) Topological plot showing the 27 genes participating in the Calcium signaling, Rap1 signaling, and PI3K-Akt signaling pathways to regulate the platelet activation. Each square represents the expression level of the gene at different time points based on the heatmap.

Thrombocytopenia is one of the symptoms caused by SFTSV infection (Yu et al., 2011). We examined the gene expression patterns in platelet activation-related pathways in HEK 293 cells infected with SFTSV to better understand the mechanism of thrombocytopenia. First, we analyzed the expression of 57 genes involved in the platelet activation pathway at different time points after infection. The study revealed that the relative expression levels of most genes were high in the early periods of infection (6 and 12 h) and lower in the later periods of infection (24 and 48 h) (Table S5, Figure S5A). Then, we used a gene enrichment network to show the enrichment characteristics of genes involved in platelet activation. By representing each enrichment term as a node and connecting pairs of nodes with kappa similarity over 0.3, five different clusters were generated by their identity and functional tags, i.e., Platelet activation, Rap1 signaling, Oxytocin signaling, Long-term depression, and Vascular smooth muscle contraction (Figure S5B). We found that 34 genes were clustered in the platelet activation MOCDE, and we further explored the expression of these 34 genes at different times post-infection using radar plots, which demonstrated that the mRNA levels of most of these genes were significantly downregulated at the latter periods (24 and 48 h) compared to the early periods (6 and 12 h) (Fig. 5A). Previous studies have shown that the Calcium signaling pathway, Rap1 signaling pathway, and PI3K-Akt signaling pathway are involved in platelet activation (Stalker et al., 2012; Li et al., 2019). The topological plots showed that GNA13 and ARHGEF12 were significantly downregulated at 48 h post-infection, and these genes inhibit the Calcium signaling pathway by regulating ROCK1 and MYL12A. We discovered that 48 h post-infection, the expression levels of GNAS, PRKACA, RAP1A, and VAMP8 were all dramatically downregulated, which inhibited Rap1 activation and transactivation. In addition, genes related to PI3K-Akt signaling pathway showed downregulation and inhibition at 48 h post-infection, such as GP1BA, PIK3CA, AKT3, and PLA2G4A (Fig. 5C). Finally, we randomly selected GNAS, ROHA, MYL12, and PLA2G4A among these 34 genes and measured their mRNA levels by qRT-PCR (Fig. 5B). These features will cause a decrease in platelet counts resulting in thrombocytopenia.

### 2.6 Protein–protein interaction network analysis of DEGs at 48 h

**Fig. 6:**
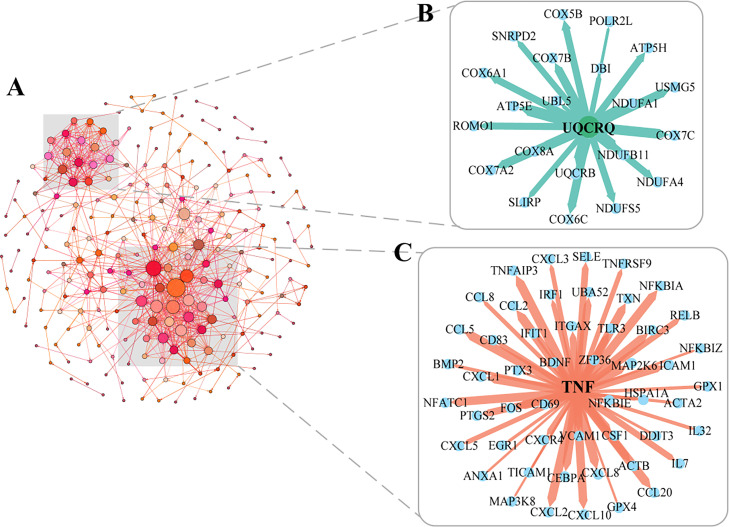
Protein–protein interaction network at 48 h (A) STRING database analysis of the differently expressed genes at 48 h. Visualization of the protein–protein interaction relationship by Gephi using a standard of combined score ≥ 0.5. Each node indicates the protein name, and the size represents the weight of the interaction. Two significant aggregation and interaction parts in the PPI network are amplified showing the different core factors in the interaction. (B) The network showing the aggregation of the gene UQCRQ as the core. (C) The cluster network key gene was TNF.

We found that the expression levels of most DEGs were most pronounced in the late stages of infection. Therefore, we used the STRING database to analyze 660 DEGs at 48 h and visualized them with Gephi, with each node representing a gene and each edge representing an interaction relationship between genes. We found that the genes at 48 h generated a network interaction graph with 236 nodes and 1634 edges, and with two significantly clustered sections (Fig. 6A). We further enlarged the two clusters to show their interacting genes, where the smaller part clustered 21 genes with UQCRQ as the core gene (Fig. 6B). The genes of these section were significantly downregulated, belonging to cytochrome c oxidase (COX) family, and have been shown to regulate the Rap1 and PI3K-Akt signaling pathways, such as COX5B, COX6A1, COX6C, COX7A2, COX7B, COX7C, and COX8A (Sobrino et al., 2010; Stalker et al., 2012; Bromberger et al., 2018; Stefanini and Bergmeier 2019). We also found a larger fraction that clustered with 51 significantly overexpressed genes centered on TNF and included cytokines such as CXCL8, CCL5, CCL2, CXCL10, CXCL1, CXCL2, and CCL20 (Fig. 6C). Moreover, we found that the top three genes that interacted most strongly with TNF were TNFAIP3, NFKBIA, and CCL2, all of which are involved in NF-κB and cytokine signaling pathways. Our study found that genes implicated in antiviral immunity were upregulated 48 h after SFTSV infection, whereas genes downregulated were primarily related to Rap1 and PI3K-Akt associated pathways of platelet activation.

## 3 Discussion

*Bunyavirales* is the largest family of arboviruses that along with the emergence of the new Phleboviruses such as SFTSV, Heartland virus (HRTV) (McMullan et al., 2012), Guertu virus (GTV) (Shen et al., 2018), Hunter island group virus (HRGV) (Gauci et al., 2015), and Lone star virus (LSV) (Swei et al., 2013) have had serious impacts on public health (Kuhn et al., 2020). Viruses are obligatory intracellular microorganisms that replicate using the machinery of the host cell (Amin and Amin 2015), and thus understanding the interaction between viruses and hosts is key to developing new antiviral therapeutic strategies.

In comparison to earlier approaches, the recently created high-throughput instrument RNA-Seq for transcriptome profiling gives substantially more accurate estimates of transcript expression levels (Wang et al., 2009). We used the Illumina NovaSeq™ 6000 platform to sequence the gene expression profiles of HEK 293 cells in different treatment groups. Analysis of randomly selected gene expression changes by qRT-PCR showed concordance with RNA-Seq results, thereby supporting the reliability of the data based on transcriptome analysis. In this study, a total of 9500 host genes were identified after infection compared to the control group, of which a total of 1145 were DEGs, accounting for 12% of the quantified genes. In addition, we found that CXCL10 was significantly upregulated in the last three stages after infection (Fig. 1A). It is a pro-inflammatory chemokine of intermediate monocytes and showed a positive correlation with IFN-α production in serum samples from patients with SFTS in the acute phase(Li et al., 2021). This confirms that SFTSV infection leads to the upregulation of CXCL10 and the related inflammatory signaling pathways. SLC family genes were also significantly up-regulated in late stages of infection, a result consistent with recent findings in THP-1 macrophages. Moreover, other studies have shown that the infection of bunyavirus RVFV can cause significant upregulation of SLC24A2 and SLCA3, suggesting that bunyavirus infection affect alterations in host ion channels, while details about SFTSV infection need to be further investigated (Pinkham et al., 2017; Yan et al., 2021).

It is well known that when a virus infects humans, innate antiviral and inflammatory responses may be triggered immediately (Basler 2017). To annotate changes in gene expression levels after SFTSV infection, we conducted out GO analysis and KEGG pathway analysis. According to GO analysis, the host established a strong immune response after being exposed to SFTSV, including NF-κB signaling, inflammatory response, immune response, and chemokine-mediated signaling (Yan et al., 2021). At 12 and 48 h after SFTSV infection, we also found that multiple DEGs were enriched in the apoptotic process, consistent with the findings of earlier proteomic analyses. As Shufen Li argued, previous research demonstrated that SFTSV infection causes monocytes to marginally trigger apoptosis (Li et al., 2020; Zhou and Yu 2021). In addition, we also observed that at 24 h post-infection, GO analysis showed significant enrichment in misfolded protein binding and unfolded protein binding, which is consistent with previous findings indicating that SFTSV infection would activate the unfolded-protein response (UPR) to eliminate misfolded proteins produced by endoplasmic reticulum (ER) stress response (Figure S2C) (Chan 2014; Zhang et al., 2019).

The KEGG results showed that, the DEGs were enriched in the NF-κB signaling pathway at 6, 24, and 48 h, showing that NF-κB signaling pathway is one of the main innate immune signaling pathways for the host resistance to SFTSV infection. Additionally, prior research has demonstrated that SFTS patients who pass away from acute illnesses have much higher TNF-α levels (Deng et al., 2012; Liu et al., 2017). In this study, DEGs were similarly enriched in the TNF signaling pathway at 12, 24, and 48 h. TNF is one of the biologically effective cytokines that activate the NF-κB signaling pathway for antiviral response, immune modulation, and apoptosis (Kalliolias and Ivashkiv 2016; Mitchell et al., 2016). These results are all consistent with the recent transcriptomic study of THP-1 macrophages infected with SFTSV, but in our study, the KEGG results of 24 h weren't enriched in the Toll-like receptor and JAK-STAT signaling pathways, and the cytokine-cytokine receptor interaction was enriched many downregulated genes, which might be caused by the different cell lines (Yan et al., 2021). Our study demonstrates that SFTSV affects these signaling pathways, and further investigation on these signaling pathways will assist in characterizing SFTSV infection and pathogenesis, identifying potential therapeutic targets, and assisting in the prevention and treatment of SFTSV infection.

Cytokines are regulated by the NF-κB signaling pathway and crucial for antiviral immunity (Hayden and Ghosh 2014). The NF-κB pathway can be activated by a variety of ligand-receptor interactions. According to prior studies, as TNFR1 is active, it binds to TNFR1-associated death domain protein (TRADD), which then attracts TNF receptor-associated factor 2 (TRAF2) and receptor-interacting protein 1 (RIP1), and the latter causes IKKβ-mediated activation of NF-κB (Sun 2012; Cildir et al., 2016). We found that SFTSV infection activates genes in the TNF signaling pathway, which in turn mediates NF-κB signaling, such as TNF, TNFAIP3, TNFRSF9, TRAF2, TRAF6, RELA, and NFKBIA. In addition, we found a significant increase in the expression levels of NLRP6, NOD2, and TRAF3. NLRP6 activates caspase-1 and NF-κB signaling pathways to produce large amounts of cytokines in antiviral immunity as previous research (Sun et al., 2015; Liu et al., 2017; Yamada et al., 2018; Li and Zhu 2020). SFTSV infection induces host cells to express IFN and ISG to further prevent viral infection. The activation of TLR3-IRFs signaling pathway is critical to induce IFN production (Chen et al., 2017; Huo et al., 2020). These data are consistent with the clinical observations of the expression kinetics of cytokines in SFTS patients, which indicate that IL-6, IL-10, CXCL10, and IFN-gamma levels are typically high during the early stage (Deng et al., 2012; Liu et al., 2017; Kwon et al., 2018). TNF-α induces an increase expression of CCL20, which chemotacticizes lymphocytes and neutrophils (Deng et al., 2012; Park et al., 2021). These high expressions levels of inflammatory cytokines indicate that the host induces an intense cytokine storm to resist the infection, which has previously been noted as a significant pathological characteristic (Sun et al., 2012). These results may provide a rational explanation for the activation of antiviral immunity induced by SFTSV infection and the excessive inflammatory response leading to host damage.

We identified 57 genes involved in the platelet activation signaling pathways, of which 34 genes were significantly downregulated at 24 and 48 h (Casel et al., 2021). We found that genes of the Calcium signaling pathway were all significantly downregulated at 24 and 48 h post-infection, such as GNA13, ARGHGEF12, ROHA, ROCK1, and MYL12A. Inhibition of this pathway will result in altered platelet shape and loss of biological function, followed by recognizing and clearing by macrophages. This is consistent with previous studies, which suggests that the spleen is the primary organ attacked by SFTSV and that macrophage clearance of virus-bound platelets in the spleen, which is one of the causes of thrombocytopenia in patients (Jin et al., 2012; Seo et al., 2021). Next, we also observed that SFTSV infection affect the activation and transactivation of the Rap1 signaling pathway, which in turn inhibit platelet activation. In particular, vesicle-associated membrane protein 8 (Vamp8) was significantly downregulated, which involved in both the direct fusion of the platelet particle envelope with the open conduit system (OCS) and the mixed fusion of the granules required for platelet activation (Ren et al., 2007; Llobet et al., 2019). In addition, the PI3K-Akt signaling pathway-related genes were expressed at lower levels in SFTSV infection 24 and 48 h, for example, GP1BA, PIK3CA, PIK3CB, AKT3, PRKG1, and PRKG2. All of these downregulated genes inhibit the activation of PLA2G4A that can activate the production of arachidonic acid (AA) metabolites thromboxane A2 (TXA2), a bioactive substance that can initiate platelet activation (Sobrino et al., 2010; Stefanini and Bergmeier 2019). GP1BA encodes a platelet surface membrane protein and can affect arterial thrombosis-mediated platelet counts (De Candia 2012; Luo et al., 2021). Additionally, severe systemic inflammation caused by SFTSV infection leads to endothelial damage resulting in increased peripheral platelet consumption, which will also lead to thrombocytopenia (Casel et al., 2021; Sun et al., 2022). Furthermore, the 48 h PPI results showed a cluster centered on UQCRQ, with most genes belonging to the cytochrome c oxidase (COX) family, and its downregulation could cause the decrease of platelet mitochondrial respiratory chain activity, such as COX5B, COX6A1, COX6C, and COX7C (Valla et al., 2006; Cimmino and Golino 2013; Iñarrea et al., 2014). Whether this is the cause of the decrease in platelet caused by SFTSV infection remains to be further studied.

Recent study in THP-1 macrophages gave the first genome-wide transcriptome analysis of host cells and SFTSV (Yan et al., 2021). Here, we explored the HEK293 cells in response to SFTSV infection, which extends the knowledge about cellular antiviral activity. By examining DEGs, we demonstrated that SFTSV infection can elicit a strong antiviral immune response in the host and that excessive inflammatory response is a core factor in host body damage. Moreover, SFTSV infection will also affect platelet production through a variety of influencing mechanisms, resulting in thrombocytopenia. This study highlights numerous crucial host biological functions that can serve as topics for future fundamental study and anti-SFTSV medication development.

## 4 Materials and methods

### 4.1 Cells culture and viruses

HEK 293 cells were culture in DEME (Gibco, USA) supplemented with 10% FBS (Gibco, USA) and kept at 37 °C with 5% CO2 (Lam et al., 2013). SFTSV Wuhan strain (GenBank accession numbers: S, KU361341.1; M, KU361342.1; L, KU361343.1) is propagated in Vero cells.

### 4.2 Sample collection and sequencing

All SFTSV-related research was carried out in accordance with institutional biosafety operating protocols in an animal biosafety level-2 laboratory (BSL-2) and physical containment level 2 laboratory (P2). SFTSV was applied at a MOI of 5 to HEK 293 cells. Three biological replicates were conducted. Total RNA of treated and MOCK cells was extracted at the designated times with Trizol (Invitrogen, USA) in accordance with the manufacturer's instructions. Total RNA of three independent samples was diluted to a concentration ≥ 1 μg and a total volume ≥ 20 μl. Oligo (dT) magnetic beads that contained poly-A tails were used to enrich mRNAs from the total RNA, then to construct the NEB libraries. The libraries were used for sequencing with the Illumina NovaSeq™ 6000 platform.

### 4.3 Enrichment analysis of DEGs

Each transcript or gene's fold change (FC) in various groups was calculated. Based on P-value ≤ 0.05 and |log2FC| ≥ 0, the genes were selected for DEGs at four time points. The DAVID was used to analyze the DEGs (https://david.ncifcrf.gov/) (Huang da, Sherman et al. 2009), and GO and KEGG pathway enrichment analysis was performed. The R package “GOplot” was adopted to show the results as a turquoise module genes plot. KEGG pathways visualization was conducted using Hiplot (https://hiplot.com.cn/) as a bubble plot.

### 4.4 Gene set enrichment analysis (GSEA) and temporal clustering analysis

GSEA interprets the different effects of collective behavior of genes on pathways by evaluating the differential expression between treatment and control groups (Reimand et al., 2019). The R language's "Clusterprofiler" package was used to perform GSEA, which was used to determine the gene set's enrichment pathways at the four time points. Statistical significance was defined as a P-value ≤0.05. Genes can be assigned to one of eight clusters using the fuzzy c-means algorithm and temporal cluster analysis of soft clustering (Futschik and Carlisle 2005). The time-dependent expression patterns of genes were shown using a noise-robust soft clustering approach carried out in R using the Mfuzz package (version 3.14, http://www.bioconductor.org/packages/release/bioc/ht ml/Mfuzz.html) (Kumar and Futschik, 2007).

### 4.5 Quantitative real-time PCR (qRT-PCR)

RNA was reverse transcribed using the PrimeScript™ RT Kit (TaKaRa, Japan) reverse transcriptase. qRT-PCR was conducted to evaluate gene expression level using the Applied Biosystems 7500 Real-Time PCR device. Specific primers to target genes were listed in Table S6.

### 4.6 Protein–protein interaction analysis

To establish a protein-protein interaction network and further screen the core target genes, DEGs were submitted to the STRING database (https://cn.string-db.org/) (Szklarczyk et al., 2019). Network diagrams were visualized using Gephi software for PPI analysis (Kauffman et al., 2014). Metascape was used to collect and draw molecular complex detection (MCODE) networks identified for individual gene lists. It could recognize tightly connected network components (https://metascape.org/gp/index.html - /main/step1) (Zhou et al., 2019).

### 4.7 Selection of immunity-associated and platelet activated genes

The PathCards database's data on immunity-related genes was searched for and downloaded (https://pathcards.genecards.org/card/lipoprotein_metabolism) (Belinky et al., 2015). The overlapped genes with those from the RNA-seq database were selected for further analysis.

### 4.8 Topological analysis

Topological plot visualization of each gene expression was based on PATHVIEW (https://pathview.uncc.edu/) (Luo et al., 2017), the gene expression fold changes from left to right in each rectangle:6, 12, 24, 48 h, and visualizing with the heatmap package in R.

### 4.9 Availability of data and materials

The original data in this study can be found in NGDC project HRA003554 (https://ngdc.cncb.ac.cn/search/?dbId=hra&q=HRA003554) with accession of 8 samples (HRX600140, HRX600139, HRX600138, HRX600137, HRX600136, HRX600135, HRX600134, HRX600133).

## CRediT authorship contribution statement

**Huimin Fu:** Methodology, Software, Formal analysis, Investigation, Writing – original draft, Writing – review & editing. **Yanhong Wang:** Conceptualization, Methodology, Formal analysis, Writing – original draft, Writing – review & editing. **Chuanfei Yuan:** Methodology, Investigation. **Yuhang Zhang:** Methodology, Investigation. **Aihua Zheng:** Methodology, Visualization, Writing – review & editing. **Zhen Zou:** Conceptualization, Resources, Supervision, Writing – review & editing, Funding acquisition. **Qianfeng Xia:** Conceptualization, Writing – original draft, Resources, Supervision, Funding acquisition.

## Declaration of Competing Interest

The authors declare that they have no known competing financial interests or personal relationships that could have appeared to influence the work reported in this paper.

## Data Availability

Data will be made available on request.

## Appendix Supplementary materials

Image, application 1

Image, application 2

Image, application 3

Image, application 4

Image, application 5

Image, application 6

Image, application 7
