# Supplementary material for: Screening core genes and signaling pathways after SFTSV infection by integrated transcriptome profiling analysis
Source: Virus Res. 2023 May 23;332:199138. doi: 10.1016/j.virusres.2023.199138 (PMC10345746; doi:10.1016/j.virusres.2023.199138)
Supplement: Supplementary file 1 [file mmc1.docx]

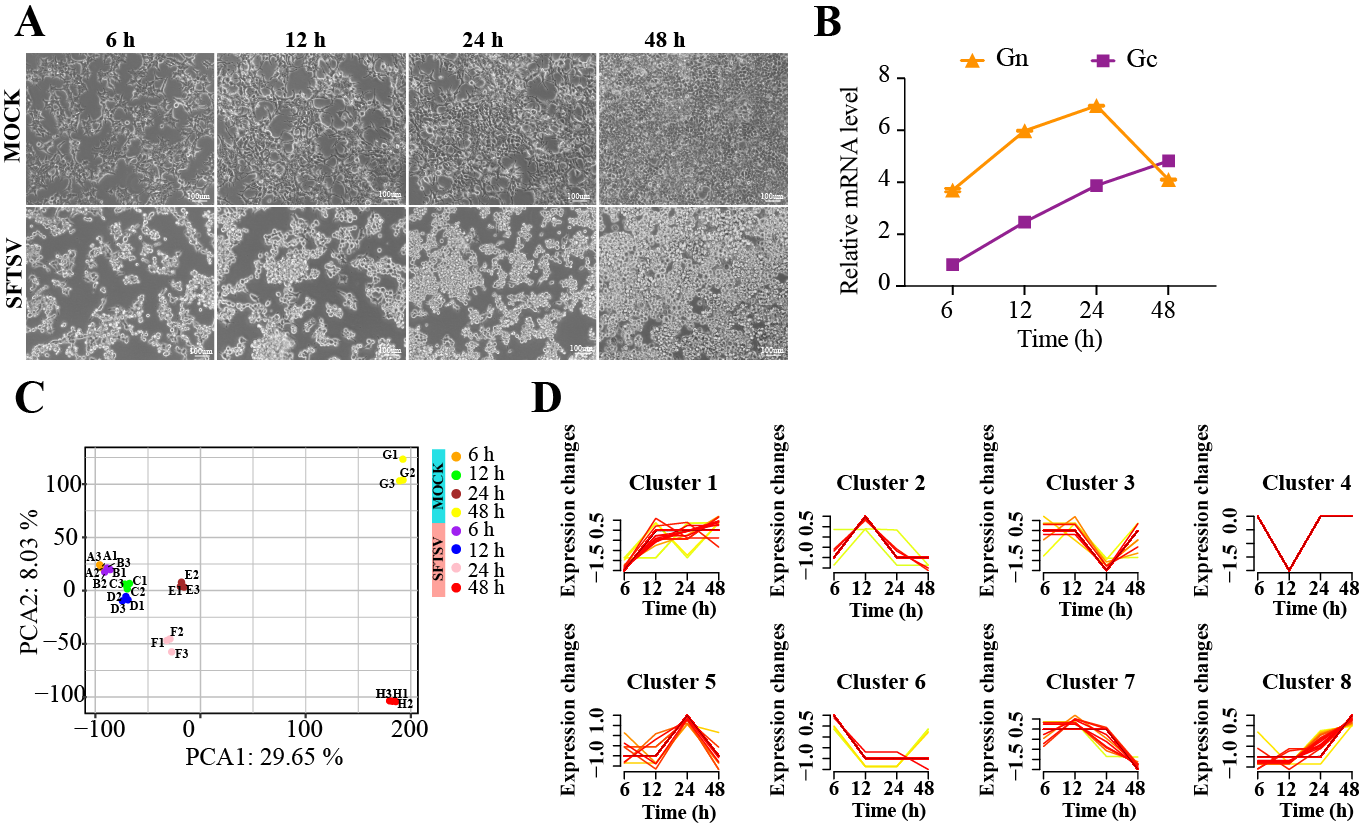


**Figure S1. Temporal clustering analysis of the DEGs**

(A) Tracking the progression of HEK 293 cells in infection and MOCK using optical microscopy. The images from left to right show the different time courses (6, 12, 24, and 48 h), to assess the viability of HEK 293. Scale bars represent 100µm. (B) SFTSV infection was verified by qRT-PCR. The SFTSV replication in cells was measured by detecting the SFTSV Gn/Gc relative mRNA levels of four-point times. Intracellular RNA levels at each time point of SFTSV infection were normalized to those in the MOCK cells. All experiments were performed at least three times, and values represent means ± the SEM. (C) Principal component analysis (PCA) of replicate samples from SFTSV and MOCK groups at different time points. To assess the duplication of RNA samples within the group, PCA was performed on the gene expression (FPKM) of all samples. Each dot represents a sample, and each color represents a group. (D) Based on the Fuzzy c-means, 1145 DEGs were clustered using mFuzz into eight distinct temporal patterns. The x-axis represents four-time points of the infection stage, indicating the 6 h, 12 h, 24 h, and 48 h, respectively. The y-axis represents the log2-transformed expression level, normalized intensity ratios in each stage. **
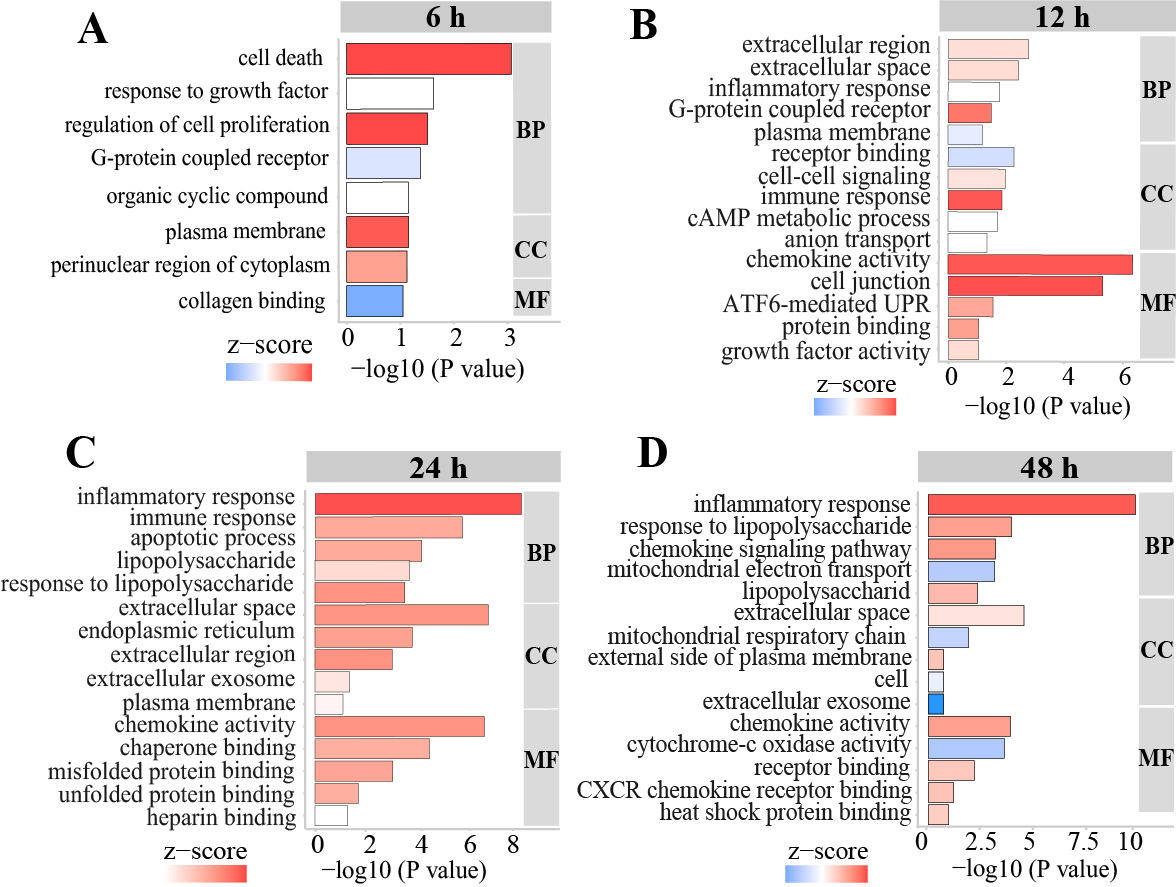
**

**Figure S2. Analysis of GO enrichment for whole DEGs at four-time point**

GO enrichment analysis of whole DEGs at 6 (A), 12 (B), 24 (C), and 48 h(D). The level of the DEGs enriched in GO categories, such as biological process (BP), cellular component (CC), and molecular function (MF). The y-axis represents the GO term and shows the top 5 GO terms of BP, CC, and MF. The x-axis shows the minus log10 scale of the P value (-log 10(P-value)). The red color indicates that most genes in this term are upregulated, and the blue color indicates most genes in this term are downregulated.


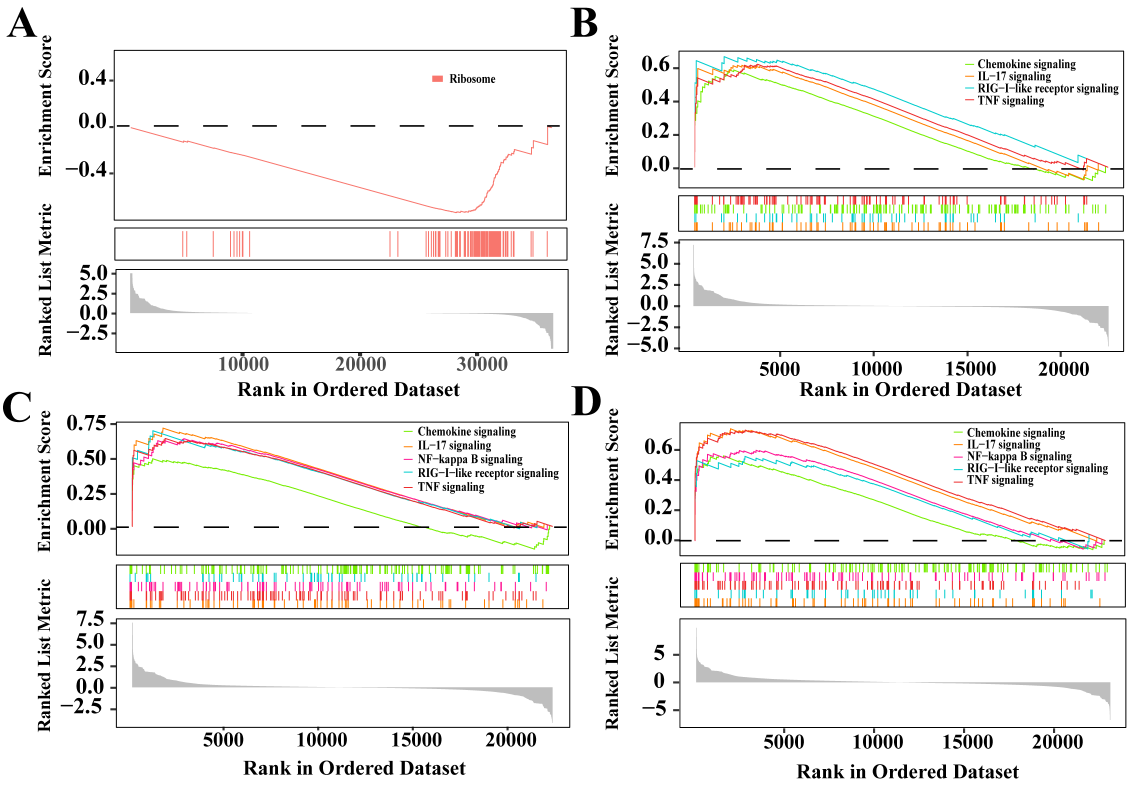


**Figure S3. GSEA analysis of the gene set of different comparison groups**

Gene set enrichment analysis (GSEA) of the gene set of different comparison groups at four-time points shows the distinct pathways enrichment at 6 (A), 12 (B), 24 (C), and 48 h (D). The top part, “Enrichment Score” refers to the enrichment degree of the gene set of the pathway at the time point. The final defined ES of the gene set is the peak in the line chart, and the gene in front of the peak is the core gene in the gene set. The middle part, various colored lines were used to mark the genes under the gene set. The bottom part, “Ranked List Metric” means the distribution of all gene rank by values, using the signal method of signal-to-noise ratio (S/N) to rank the log2FC of each gene. “Rank in Ordered Dataset” indicates the rank value distribution map of all genes.


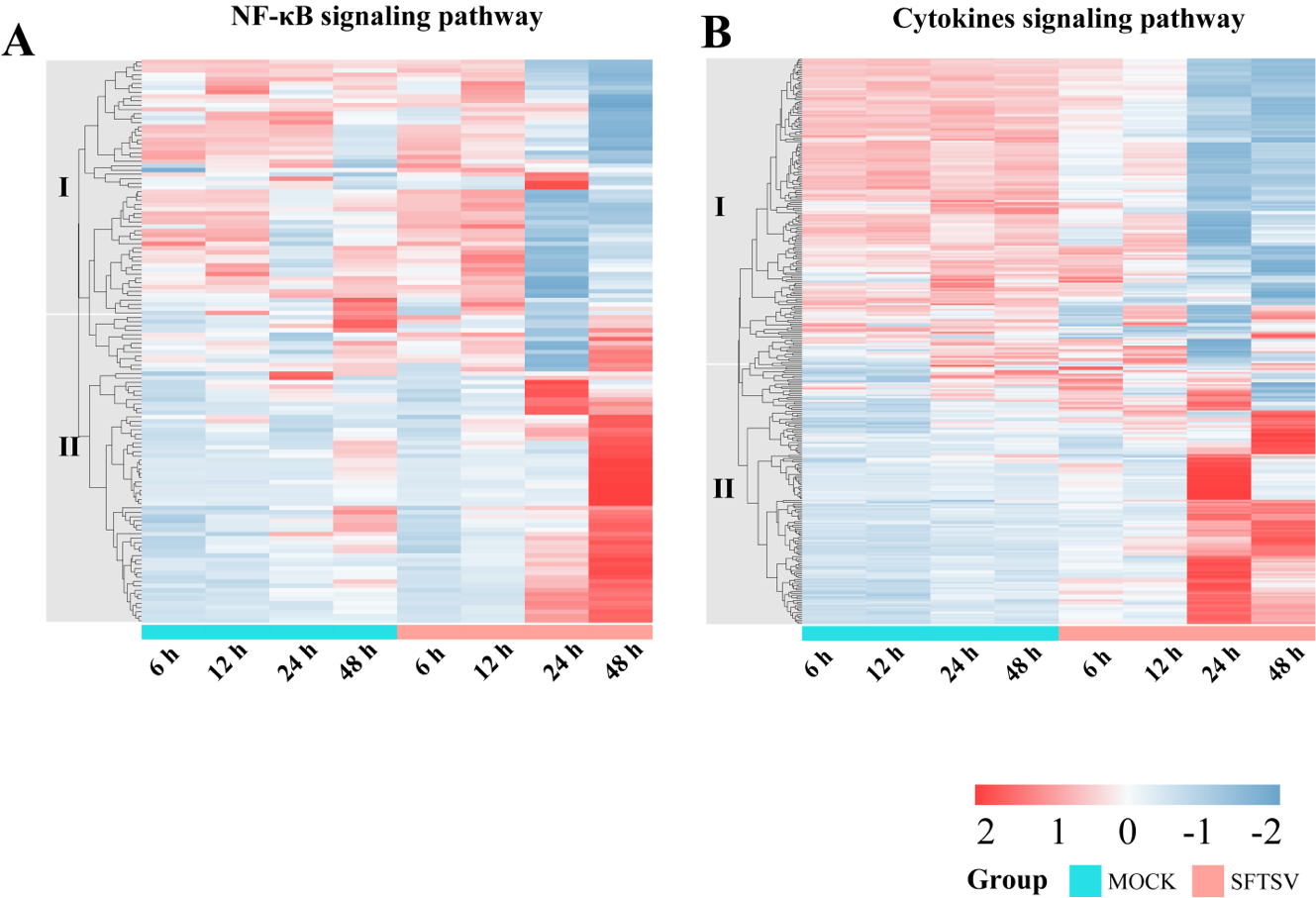


**Figure S4. Heatmap analysis the antiviral immune pathway at four-point times**

Heatmap and clustering analysis of the relative expression of the signaling pathway-related genes at four-time points. Red indicates high expression and blue represents low expression, -1,0 and 1 represent Z-score. (A), NF-𝜅B signaling pathway, and (B), Cytokines signaling pathway. Each signaling pathway is shown with two clusters, Cluster I and Cluster II include genes whose expression was significantly down-regulated and up-regulated at 48 h post-infection relative to 6, 12, and 24 h, respectively.


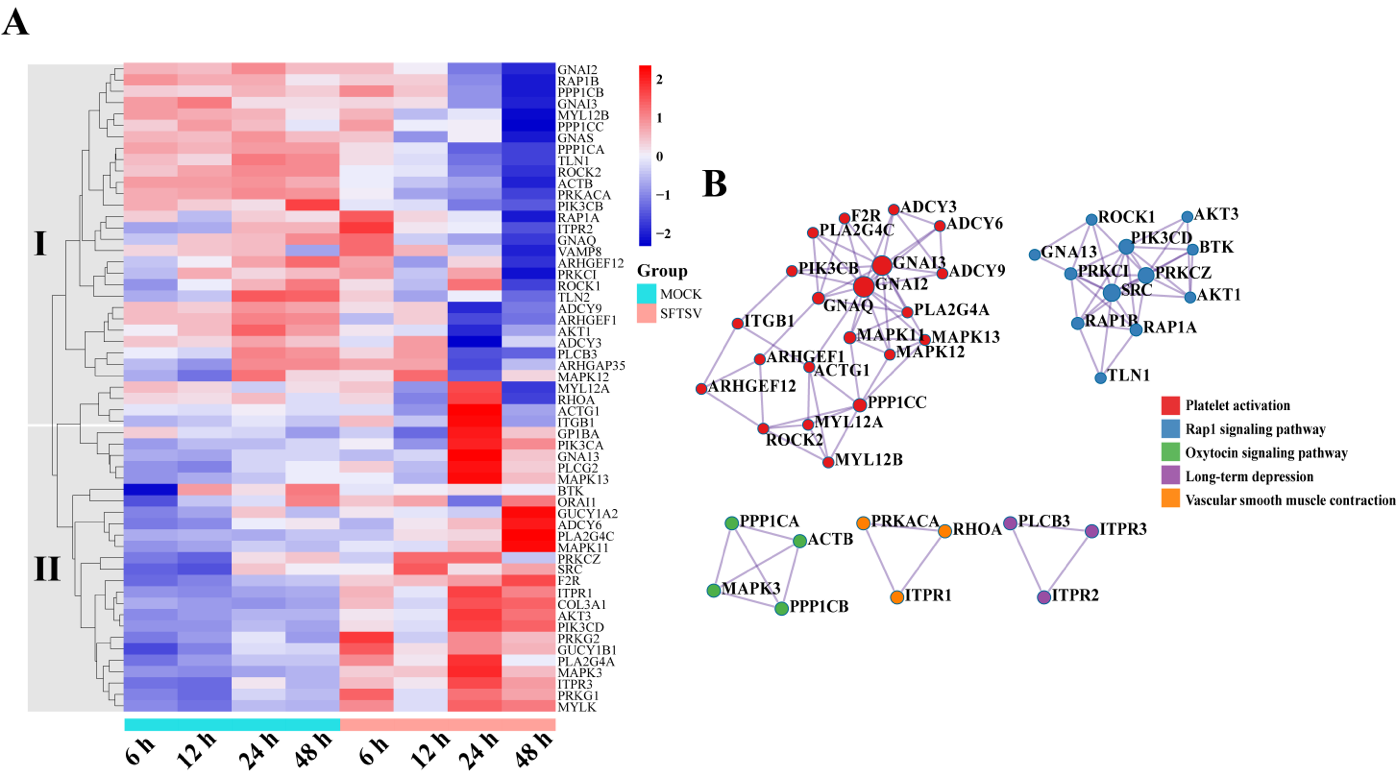


**Figure S5. SFTSV infection causes the platelet related genes down-regulation**

(A) Heatmap shows the whole 57 genes involved in the platelet activation pathway. (B) Metascape visualization of the interactome network formed by 57 genes, where the five MCODE complexes are colored and labeled according to their identities, all nodes represent the gene's name.
